# Artificial Intelligence in Fetal Growth Restriction Management: A Narrative Review

**DOI:** 10.1002/jcu.23918

**Published:** 2025-01-29

**Authors:** Ugo Maria Pierucci, Gabriele Tonni, Gloria Pelizzo, Irene Paraboschi, Heron Werner, Rodrigo Ruano

**Affiliations:** ^1^ Department of Pediatric Surgery “V. Buzzi” Children's Hospital Milan Italy; ^2^ Department of Obstetrics & Neonatology, and, Researcher Università degli Studi di Modena e Reggio Emilia—Sede di Reggio Emilia Reggio Emilia Italy; ^3^ Department of Biomedical and Clinical Science University of Milano Milan Italy; ^4^ Biodesign Lab Dasa/PUC‐Rio Pontificia Universidade Catolica Rio de Janeiro Rio de Janeiro Brazil; ^5^ Division of Maternal‐Fetal Medicine, Department of Maternal and Fetal Medicine, Obstetrics and Gynecology University of Miami, Miller School of Medicine Miami Florida USA

**Keywords:** artificial intelligence, fetal biometry, fetal growth restriction, machine learning, predictive analytics, prenatal diagnosis, ultrasound imaging

## Abstract

This narrative review examines the integration of Artificial Intelligence (AI) in prenatal care, particularly in managing pregnancies complicated by Fetal Growth Restriction (FGR). AI provides a transformative approach to diagnosing and monitoring FGR by leveraging advanced machine‐learning algorithms and extensive data analysis. Automated fetal biometry using AI has demonstrated significant precision in identifying fetal structures, while predictive models analyzing Doppler indices and maternal characteristics improve the reliability of adverse outcome predictions. AI has enabled early detection and stratification of FGR risk, facilitating targeted monitoring strategies and individualized delivery plans, potentially improving neonatal outcomes. For instance, studies have shown enhancements in detecting placental insufficiency‐related abnormalities when AI tools are integrated with traditional ultrasound techniques. This review also explores challenges such as algorithm bias, ethical considerations, and data standardization, underscoring the importance of global accessibility and regulatory frameworks to ensure equitable implementation. The potential of AI to revolutionize prenatal care highlights the urgent need for further clinical validation and interdisciplinary collaboration.

## Graphical abstract

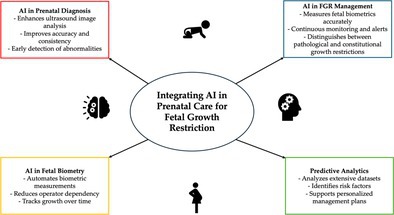

## 1 Introduction

Fetal growth restriction (FGR) is defined as the underdevelopment of the fetal potential to grow in utero and can be classified as early (< 32 weeks of gestation) and late FGR (> 32 weeks of gestation). Clinically, one of the most common clinical diagnostic clusters of the condition used is an ultrasound estimated fetal weight (EFW) below the 10th percentile for a given gestational age (GA) [1, 2].

Very recent publications have demonstrated that ultrasound biometry (with or without the implementation of Doppler assessment) is still a poor predictor of histopathologic placental abnormalities associated with and seen in cases of placental vascular insufficiency [3, 4]. Indeed, although the epidemiology of FGR recognizes multifactorial causes, placental vascular insufficiency is one of the most important underlying causes leading to FGR and its related adverse perinatal outcome [5].

An adverse perinatal outcome, with an increase in both perinatal mortality and morbidity, is particularly observed in FGR fetuses with recognized pathological causes compared to constitutionally small fetuses [6, 7, 8].

There are different guidelines and proposals to manage perinatally babies affected by FGR [9]. We hypothesize that Artificial Intelligence (AI) may help uniformize prenatal diagnosis and perinatal management in the future and define the best approach for these patients.

The integration of AI in prenatal care, particularly in prenatal diagnosis and the management of FGR, has the potential to transform the field by enhancing diagnostic accuracy, improving monitoring, and enabling personalized treatment plans [10, 11].

A report from the World Health Organization (WHO), published in 2022, reported that over 20 million of newborns have a birth weight of less than 2500 g, defined as low birth weight (LBW). It is noteworthy to underline that in most cases (around 90%), LBW babies are delivered in southern Asia and sub‐Saharan Africa, and of those neonates who die, around 80% were born before 37 weeks of gestation or were small for gestational age (SGA). In this concern, the WHO has delivered a guideline based on three different types of actions summarizable in: prevention, treatment of prematurity complications, and family engagement [12, 13, 14, 15, 16]. It can be postulated that targeted developed AI applications could help improve the obstetric detection of fetuses affected by FGR, ameliorating antenatal management and thus perinatal care as well as early and late outcomes. AI could offer the potential to revolutionize the management of FGR by enabling earlier and more accurate identification of at‐risk pregnancies. By integrating advanced algorithms, AI can analyze complex patterns in maternal, fetal, and placental data that traditional methods may overlook. This could lead to more precise risk stratification, timely interventions, and improved perinatal outcomes. Furthermore, AI's capacity to optimize resource allocation and reduce operator dependency underscores its critical role in advancing prenatal care. This narrative review addresses applications' advancements, challenges, and future directions associated with AI in FGR management, including algorithmic bias, ethical implications, and the need for standardized, high‐quality data sets. Furthermore, it emphasizes the importance of fostering global accessibility and establishing robust regulatory frameworks to ensure equitable and effective implementation. By tackling these issues, the transformative potential of AI in prenatal care can be fully realized, paving the way for advancements in clinical validation and fostering interdisciplinary collaboration.

## 2 Enhancing Prenatal Diagnosis With AI

AI has made significant strides in prenatal diagnosis, mainly through ultrasound image analysis and genetic screening. Traditional ultrasound diagnostics are highly dependent on the skills and experience of the sonographer, leading to variability in detection rates. AI algorithms, however, provide a significant advantage by analyzing ultrasound images with greater accuracy and consistency. Studies such as those by Liu et al. [10] in 2024 and Wenjia Guo et al. [17] in 2023 have shown that AI systems can detect congenital heart defects, neural tube defects, and other structural abnormalities earlier and more accurately than conventional methods. This early detection is crucial for timely interventions that can mitigate potential complications during pregnancy and post‐birth [10, 17].

Non‐Invasive Prenatal Testing (NIPT) also benefits from AI advancements. AI algorithms analyze cell‐free fetal DNA in maternal blood samples, improving the detection of chromosomal abnormalities such as trisomy 21 (Down syndrome), trisomy 18 (Edwards syndrome), and trisomy 13 (Patau syndrome). This method is less risky than invasive procedures like amniocentesis [18, 19].

Predictive analytics powered by AI are essential for assessing the risk of pregnancy‐related complications, such as gestational diabetes and preterm birth. AI models can analyze extensive data sets from electronic health records to identify patterns and risk factors that may not be immediately apparent to clinicians [20, 21]. This capability allows for timely interventions and better management of high‐risk pregnancies [20, 21].

## 3 Application of Machine Learning in Prenatal Care

Machine learning, a subset of AI, plays a critical role in prenatal care by enabling the development of predictive models and enhancing diagnostic precision. Machine‐learning algorithms can process vast amounts of data, including ultrasound images, genetic information, and electronic health records, to identify patterns and accurately predict outcomes [22, 23].

For example, supervised machine‐learning algorithms are trained on labeled data sets, where the input data and corresponding output are known. This training allows the algorithms to learn the relationship between the data and the desired outcome. In prenatal care, supervised machine learning can be used to predict the risk of complications by analyzing historical patient data and identifying key risk factors [22].

On the other hand, unsupervised machine learning deals with unlabeled data and aims to identify hidden patterns or clusters within the data. In the context of prenatal care, unsupervised learning can be used to group patients with similar characteristics or identify new risk factors for pregnancy complications. For instance, clustering algorithms can segment patients based on their ultrasound image patterns, helping clinicians identify those who may require closer monitoring [22].

Deep learning, a subset of machine learning, involves neural networks with multiple layers that can learn complex patterns in large data sets. Deep‐learning algorithms have shown remarkable success in medical image analysis, including prenatal ultrasound images. Convolutional neural networks (CNNs), a type of deep learning algorithm, can automatically detect and classify abnormalities in ultrasound images with high accuracy, reducing the reliance on operator expertise and improving diagnostic consistency [22].

Integrating machine learning and deep learning in prenatal care not only enhances diagnostic accuracy but also enables the development of personalized treatment plans. By analyzing individual patient data and identifying unique risk factors, these algorithms can help clinicians tailor interventions to the specific needs of each patient, ultimately improving maternal and fetal outcomes [23]. In a commentary to a paper by Kegel et al. [24] also Tonni and Grisolia advocated that the time to include AI to assist prenatal ultrasound diagnosis has come [25].

## 4 AI in Fetal Biometry

Fetal biometry involves measuring various fetal parameters to assess growth and development. AI can significantly enhance these measurements accuracy, reproducibility, and efficiency, which are crucial for monitoring fetal health.

The primary biometric parameters used in fetal growth assessment include:

- Biparietal Diameter (BPD): Measurement of the fetal head width.
- Head Circumference (HC): Circumferential measurement of the fetal head.
- Abdominal Circumference (AC): Measurement around the fetal abdomen.
- Femur Length (FL): Length of the fetal femur bone [26].

AI algorithms can automate the segmentation of fetal structures in ultrasound images, reducing operator dependency and increasing measurement consistency [27]. For instance, automated segmentation tools powered by deep learning can delineate the boundaries of fetal organs and bones with high precision, facilitating accurate biometric assessments [28].

Recent advancements further illustrate the depth of AI's potential in fetal biometry. Ghelich Oghli et al. [29] developed an innovative deep convolutional network architecture that predicts fetal biometry with remarkable precision, streamlining routine clinical workflows. Venturini et al. [30] introduced a comprehensive AI‐powered framework capable of estimating fetal biometrics from 20‐week ultrasound scans in real‐time, underscoring its scalability and applicability in diverse clinical settings. Similarly, Han et al. [31] evaluated automated software for mid‐trimester biometry measurements, demonstrating that AI can match the precision of manual methods while significantly reducing variability and time requirements. Expanding beyond routine measurements, Zeidan et al. [32] showcased an AI‐driven approach for diagnosing and analyzing the texture of the fetal liver and placenta in cases of fetal growth restriction, highlighting AI's utility in addressing complex pathological conditions. Meanwhile, Płotka et al. [33] proposed BabyNet, a residual transformer module designed to predict birth weight from fetal ultrasound videos, exemplifying how AI can effectively utilize dynamic longitudinal data. Finally, Matthew et al. [34] explored a groundbreaking paradigm in real‐time anomaly detection during fetal ultrasound scans, emphasizing AI's capability to redefine diagnostic processes with unprecedented accuracy and immediacy. These advancements collectively demonstrate AI's transformative role in fetal biometry, enabling precise monitoring of fetal health while paving the way for more consistent and efficient prenatal care.

### 4.1 AI in the Prenatal Diagnosis of FGR

AI technologies have shown promise in enhancing the accuracy and efficiency of FGR diagnosis. These technologies can analyze vast amounts of data from various sources, including medical imaging and electronic health records, to identify patterns and predict outcomes [11, 35].

Supervised learning algorithms trained on labeled data sets can classify whether a fetus is at risk of FGR based on input features such as maternal characteristics, fetal measurements, and Doppler indices [36, 37, 38]. For example, Zhang and colleagues [28] proposed an automatic image quality assessment scheme based on multitask learning to improve fetal sonographic image quality control. They found that their method could accurately identify essential anatomical structures in fetal sonographic images, ensuring that the images met quality standards for biometric measurements and anomaly diagnosis. Using three convolutional neural networks, their approach demonstrated competitive performance in segmentation and diagnosis while significantly reducing the time required for quality assessment to less than a second, thereby offering a practical solution for streamlining labor‐intensive sonographic evaluations.

These algorithms can outperform traditional statistical methods by identifying complex, non‐linear relationships in the data [39].

Unsupervised learning techniques, such as clustering, can reveal novel patterns in fetal growth data that might not be apparent through conventional methods. For example, unsupervised clustering has been used to identify subgroups of fetuses with distinct growth trajectories, which can provide insights into different etiologies of FGR [39].

### 4.2 AI in the Perinatal Management of FGR

FGR poses significant risks to fetal development and long‐term health outcomes. AI enhances the early detection and diagnosis of FGR by improving the precision of ultrasound imaging. AI algorithms can measure fetal biometrics with high accuracy, allowing for the early identification of growth restrictions. This capability is crucial for implementing timely and appropriate interventions [40].

Another critical area where AI excels is the continuous monitoring of fetal growth. AI‐powered tools can analyze data from wearable devices and ultrasound scans, providing real‐time updates and alerts to healthcare providers. This continuous tracking helps distinguish between pathological growth restrictions and constitutionally small fetuses, informing appropriate management strategies [41, 42].

Predictive analytics also play a pivotal role in managing pregnancies affected by FGR. AI models stratify the risk of adverse outcomes by considering multiple factors, including maternal health, fetal condition, and placental function. This risk assessment helps clinicians develop personalized management plans that address each pregnancy's specific needs and improve overall outcomes [37].

AI tools present significant strengths in the management of FGR. They enhance diagnostic accuracy by automating complex tasks such as fetal biometry and Doppler analysis, reducing operator dependency, and increasing measurement consistency [43]. Furthermore, AI's ability to analyze large data sets and detect patterns allows for early identification of at‐risk pregnancies and personalized treatment planning [44]. These tools also offer scalability, making advanced diagnostic capabilities accessible to underserved regions with limited expertise [10].

However, limitations in the application of AI in FGS management remain. The performance of AI algorithms is highly dependent on the quality and diversity of training data sets, which may not always reflect real‐world variability [45]. Algorithmic bias poses a significant challenge, potentially leading to disparities in care [44]. Moreover, the integration of AI into clinical practice requires substantial infrastructural and technical investments, which may not be feasible for low‐resource settings [46]. Regulatory hurdles and the lack of standardized evaluation frameworks further complicate the widespread adoption of AI tools [47]. Addressing these limitations is essential to fully realize the potential of AI in improving outcomes for FGR.

Moreover, current AI tools do not fully address several critical areas in FGR management. For instance, while AI excels in pattern recognition and risk stratification, it lacks the capacity for causal inference, limiting its utility in understanding the underlying mechanisms of FGR [11, 48]. Additionally, current models often rely on static data sets, which do not account for the dynamic nature of pregnancy and fetal development [49]. Integrating longitudinal data and real‐time monitoring could significantly enhance AI's predictive accuracy and clinical utility.

Future advancements should focus on overcoming these gaps by developing adaptive algorithms capable of integrating diverse data streams, such as genetic markers, environmental factors, and longitudinal imaging data. Expanding the scope of AI applications to include real‐time decision support and personalized therapeutic interventions will also be crucial. Moreover, fostering multidisciplinary collaborations among clinicians, data scientists, and ethicists can ensure that AI tools are both clinically relevant and ethically sound. Addressing these challenges will pave the way for a more holistic and effective approach to FGR management.

## 5 Integrating AI for Improved Clinical Decision‐Making

AI‐powered decision support systems enhance clinical decision‐making by integrating data from various sources and providing evidence‐based recommendations. These systems improve the efficiency and accuracy of clinical decisions [50, 51]. Additionally, AI can automate the generation of comprehensive reports on fetal growth and risk assessments, improving documentation and communication among healthcare providers [11].

However, successfully integrating AI into clinical practice faces challenges, particularly regarding data quality, ethical and legal issues, and clinical acceptance. The effectiveness of AI models depends on the quality and comprehensiveness of the data used for training and analysis [52]. Ethical and legal concerns, primarily concerning data privacy, informed consent, and potential biases in AI algorithms, must be addressed to ensure the safe and equitable use of AI technologies [40, 51].

## 6 Ethical Considerations

The integration of AI in prenatal care raises important ethical considerations that must be addressed to ensure safe and equitable use. The integration of AI involves collecting and analyzing sensitive personal health data, necessitating stringent measures to protect patient privacy and confidentiality. Ensuring informed consent is crucial; patients must be fully informed about how their data will be used, the benefits and risks of AI applications, and their rights to opt out of AI‐based diagnostics and treatments [44].

The regulation of AI tools in health care is critical to ensure their safety, efficacy, and public trust. Regulatory frameworks should address key concerns such as data privacy, accountability, ethical biases, and security while remaining adaptable to accommodate rapid technological advancements. These frameworks must balance promoting innovation with establishing rigorous AI development and deployment standards. For instance, data privacy regulations like GDPR in Europe set critical precedents for managing sensitive health information, while policies on software as a medical device provide pathways for approval and clearance. Flexible yet comprehensive regulation can mitigate risks, encourage equitable implementation, and foster global trust in AI‐driven solutions for health care [53, 54].

Another significant ethical issue is the potential for bias in AI algorithms. If AI systems are trained on data sets not representative of diverse populations, they may perpetuate or even exacerbate existing health disparities. To ensure fair and unbiased healthcare delivery, it is essential to develop AI models that are inclusive and consider a wide range of demographic and socio‐economic factors [44].

Transparency and accountability in AI decision‐making processes are also critical. Healthcare providers should be able to understand and explain AI‐driven recommendations and decisions to patients, fostering trust and acceptance of AI technologies. Furthermore, establishing clear regulatory frameworks and guidelines for developing and using AI in health care can help address legal and ethical challenges and ensure that AI applications meet high standards of safety, efficacy, and fairness [44].

## 7 Future Directions

The future of AI in prenatal care looks promising. Continuous advancements in AI algorithms expected to enhance the accuracy and reliability of AI applications in prenatal diagnosis and FGR management. Developing more sophisticated models that handle complex and nuanced data will improve diagnostic and predictive capabilities, leading to better clinical outcomes [20, 41].

Integrating AI tools with Electronic Health Records (EHR) systems will facilitate seamless data sharing and improve overall management of prenatal care and FGR. This integration will enable comprehensive analyses that leverage all available data, enhancing clinical decision‐making and patient care [51].

Man creates AI, and in this line, it should be regarded. Engineers and physicians must be called to work side‐by‐side to develop clinically useful tools that would be able, theoretically, to either enhance the diagnostic processes and reduce associated healthcare costs. The validation process is one of the most striking and dramatic issues in developing AI software in healthcare. Level 2 and/or Level 3, used before a specific medications or vaccine release, should also be applied to the release of such applications after validation in perinatal medicine. Notwithstanding, the validation process should be carried out at university‐tertiary referral centers with many patients managed, and external independent statistical services should treat data in the same way a double‐check randomized control trial (RCT) is produced. Neural artificial networks (NANs) with low‐cost internet lines will help link all types of healthcare providers and patients together, improving the link between local hospitals and communities and improving the use of telemedicine. With the use of AI, big meta‐data generated worldwide can be cropped together to share previous experiences conducted in monitoring pregnancy complications (e.g., gestational diabetes or preterm babies) by discharge telecare programs [55, 56, 57, 58, 59, 60, 61, 62, 63]. In this rapidly evolving scenario, public or private health authorities will be responsible for a new policy strategy that will further and significantly integrate digital technological advancements with a more patient‐centered medicine that needs to rely more on clinical evidence [64].

Efforts should also be made to make AI‐based management tools accessible globally, particularly in low‐resource settings that can significantly impact maternal and fetal health outcomes. To achieve this, it is essential to address barriers such as data quality, infrastructure limitations, and regulatory frameworks. Developing robust eHealth environments, investing in connectivity, and training healthcare professionals in AI applications are crucial steps for ensuring that these tools can be effectively deployed and scaled in diverse healthcare systems, including those in low‐and‐middle‐income countries (LMICs) [65]. Developing cost‐effective AI solutions and ensuring their availability and usability in diverse healthcare settings will promote global health equity and improve outcomes for pregnant women and their babies.

## 8 Conclusions

AI applications in prenatal diagnosis and FGR management are transforming prenatal care by enhancing early detection, monitoring, and treatment planning. As AI technology continues to evolve, its integration into clinical practice promises improved outcomes for pregnancies affected by growth restrictions, offering a new era of precision medicine in obstetrics.

## Author Contributions

**Ugo Maria Pierucci, Gabriele Tonni, and Rodrigo Ruano:** conceptualization. **Ugo Maria Pierucci, Gabriele Tonni, Gloria Pelizzo and Rodrigo Ruano:** methodology, validation, and resources. **Ugo Maria Pierucci, Gabriele Tonni, Irene Paraboschi, Heron Werner, Gloria Pelizzo, and Rodrigo Ruano:** investigation. **Gabriele Tonni, Ugo Maria Pierucci, and Irene Paraboschi:** data curation, writing – original draft preparation, visualization, and project. **Rodrigo Ruano:** writing – review and editing. All authors have read and agreed to the published version of the manuscript.

## Ethics Statement

The authors have nothing to report.

## Consent

The authors have nothing to report.

## Conflicts of Interest

The authors declare no conflicts of interest.

## Data Availability

Data is available upon request from authors.
